# Medical education and training within congenital cardiology: current global status and future directions in a post COVID-19 world

**DOI:** 10.1017/S1047951121001645

**Published:** 2021-04-12

**Authors:** Colin J McMahon, Justin T Tretter, Andrew N Redington, Frances Bu’Lock, Liesl Zühlke, Ruth Heying, Sandra Mattos, R Krishna Kumar, Jeffrey P Jacobs, Jonathan D Windram

**Affiliations:** 1 Department of Paediatric Cardiology, Children’s Health Ireland at Crumlin, Dublin, Ireland; 2 School of Medicine, University College Dublin, Belfield, Dublin 4, Ireland; 3 The Heart Institute, Cincinnati Children’s Hospital Medical Center, Cincinnati, OH, USA; 4 Department of Pediatrics, University of Cincinnati College of Medicine, Cincinnati, OH, USA; 5 Department of Paediatric Cardiology, East Midlands Congenital Heart Centre, University Hospitals of Leicester NHS Trust, Leicester, UK; 6 Division of Paediatric Cardiology, Department of Paediatrics, Red Cross War Memorial Children’s Hospital, University of Cape Town, Cape Town, South Africa; 7 Division of Cardiology, Department of Medicine, Groote Schuur Hospital University of Cape Town, Cape Town, South Africa; 8 Department of Paediatric Cardiology, Leuven, Belgium; 9 Department of Paediatric Cardiology, Royal Portuguese Hospital, Recife, Brazil; 10 Amrita Institute of Medical Sciences and Research Centre, Kochi, Kerala, India; 11 Congenital Heart Center, Division of Thoracic and Cardiovascular Surgery, Department of Surgery, University of Florida, Gainesville, Florida, USA; 12 Department of Cardiology, Mazankowski Heart Institute, University of Alberta, Edmonton, Alberta, Canada

**Keywords:** Adult congenital heart disease, congenital cardiology, congenital heart disease, education, paediatric cardiology, training

## Abstract

Despite enormous strides in our field with respect to patient care, there has been surprisingly limited dialogue on how to train and educate the next generation of congenital cardiologists. This paper reviews the current status of training and evolving developments in medical education pertinent to congenital cardiology. The adoption of competency-based medical education has been lauded as a robust framework for contemporary medical education over the last two decades. However, inconsistencies in frameworks across different jurisdictions remain, and bridging gaps between competency frameworks and clinical practice has proved challenging. Entrustable professional activities have been proposed as a solution, but integration of such activities into busy clinical cardiology practices will present its own challenges. Consequently, this pivot towards a more structured approach to medical education necessitates the widespread availability of appropriately trained medical educationalists, a development that will better inform curriculum development, instructional design, and assessment. Differentiation between superficial and deep learning, the vital role of rich formative feedback and coaching, should guide our trainees to become self-regulated learners, capable of critical reasoning yet retaining an awareness of uncertainty and ambiguity. Furthermore, disruptive innovations such as “technology enhanced learning” may be leveraged to improve education, especially for trainees from low- and middle-income countries. Each of these initiatives will require resources, widespread advocacy and raised awareness, and publication of supporting data, and so it is especially gratifying that *Cardiology in the Young* has fostered a progressive approach, agreeing to publish one or two articles in each journal issue in this domain.

Given the extraordinary, and measurable, improvements in our field of paediatric and congenital cardiac care that have occurred as a result of our investment in research and development over the past five decades, it is perhaps surprising that the way in which we train the next generation of clinicians and researchers has in some ways changed relatively little over that time. However, the past few years have seen increasing attention to how the training and education of congenital cardiologists are structured and delivered.^1-9^ This evolution is particularly important given the fears and stresses commonly experienced by trainees during congenital cardiology training.^10^ Although clear guidelines and curricula now exist for trainees,^11^ the approach to optimising performance of the trainers, most of whom have had no formal training in medical education, remains far less structured. Nonetheless, increasing numbers of us are undergoing formal educationalist training in an effort to optimise delivery of educational material and skills training to trainees.^12^ Several fundamental questions remain:

- How well are we educating congenital cardiology trainees?
- Are we getting the balance right between experiential and academic learning?
- Is competency-based medical education^13,14^ fit for purpose and does it effectively fulfil its goals in congenital cardiology training?
- How do we bridge the gaps between competencies and clinical practice, and how will we adopt entrustable professional activities into congenital cardiology training?
- Do we provide didactic lectures or problem-based learning or a hybrid of both?
- How do we provide assessment *for* learning?
- Do we provide programmatic assessment and a portfolio-based evaluation?
- What is the role of simulation within our programmes?
- How do we provide feedback to trainees?
- And, how do we foster a culture of self-regulated learning, supported by coaching, especially in postgraduate or continuing medical education?

Lastly, educational delivery is an organic process, evidenced by the rise of technology enhanced learning such as the development of dedicated learning websites (e.g., Heart University, Congenital Heart Academy, and World University for Pediatric and Congenital Heart Surgery), and the proliferation of webinars driven by the current climate of Coronavirus Disease 2019 (COVID-19). Where do these technological enhancements fit into the structure of future congenital cardiology education?

In this review, we will attempt to answer some of these questions, assessing current approaches and describing the potential for modernising and improving congenital cardiac education in the future. Because of our personal experiences, and the preponderance of published literature, we will use the systems in North America, United Kingdom, Ireland, and other European countries as templates for discussion, but several of the concepts could be applicable, and likely may prove necessary, in all training programmes around the world, notwithstanding the specific competencies required in specific parts of the world.

## Current status of training and education

### Paediatric cardiology

While subspecialty recognition is not universal around the world, paediatric cardiology training is becoming formalised almost everywhere (Table 1). In the United States of America, paediatric cardiology fellowships typically follow a 3-year paediatric residency, and then trainees undertake a 3-year core paediatric cardiology fellowship, with specific training guidelines,^1,2^ followed by a fourth specialty year if the trainee chooses a further area of subspecialty training (i.e., non-invasive imaging, cardiac catheterisation, electrophysiology, heart failure/transplantation).^3-7^ In Ireland, cardiology specialist registrars typically undergo a 5-year training programme, as an accredited speciality with a well-developed curriculum, as part of an All Island Congenital Heart Network training programme. There is marked variability in the number of fellows between different programmes. Some large programmes in the United States of America may have up to 18 core training fellows, whereas other programmes in the United States of America, and most in Ireland, United Kingdom, and Europe, have 4–5 cardiology fellows or registrars, and in smaller programmes and those in less resourced countries there may be 1 or 2. While comprehensive training curricula and guidelines are in place in the United States America and Europe, and largely fulfilled, concern exists that the appropriateness of those curricula could be improved. For example, there is marked variation in the exposure of trainees to adult congenital heart disease in the 60 United States paediatric and 231 adult cardiology programmes (https://www.nrmp.org/fellowships/pediatrics-specialities-match https://www.nrmp.org/fellowship-match-data/).^15^ Some authors have highlighted that given the majority of paediatric cardiologists work in an outpatient setting, greater exposure to ambulatory outpatient care is necessary during fellowship.^16^

**Table 1.** Comparison of training fellowships/systems across different countries.

Country	Training (yrs)	Advanced Training^*^	Research^**^	Exit/Certification
US	3–4^Φ^	Yes (4^th^ yr)	Yes	Board Certification(ABP)
UK	5	Yes (1–2 yrs)	Yes	CCT
Ireland	5	Mixed	Yes	CSCST
Belgium	3	Mixed	Yes	Paediatrics certification
Brazil	2–4^ω^	Mixed	Yes	National Certification
India	3	International	Yes	DNB or DM (2 streams)
South Africa	3	International	Yes	Certificate in PC

Abbreviations: ABP = American Board of Pediatrics; AEPC = Association for European Paediatric and Congenital Cardiology; CCT = Certificate Completion of Training; CSCST = Certificate of Satisfactory Completion Specialist Training; DM = Doctorate of Medicine; DNB = Diplomate of the National Board; PC = Paediatric Cardiology; US = United States of America; UK = United Kingdom; yr = years.

* Advanced Training: advanced subspecialist training is available in the trainees’ home country, or trainees go to international centre or mixed hybrid options.

** Research: research is part of the training curriculum.

Φ Optional fourth-year subspecialist training.

ω Optional third and fourth-year sub-specialist training at home or abroad

In the United Kingdom, paediatric cardiology training is accredited as a full speciality of its own and also encompasses adult congenital heart disease training depending on the training centre and the trainee. The precise curriculum is currently in revision with the General Medical Council due to the exigencies of the “Shape of Training Review” https://www.gmc-uk.org/-/media/documents/shape-of-training-final-report_pdf-53977887.pdf. It is likely to be similar to the current version with some changes in nomenclature (“competence” will change to “capability”) and emphasis “to produce doctors with the generic professional and specialty specific capabilities needed to manage children with acquired heart disease and patients with congenital heart disease (CHD) presenting at any age; in-utero, in childhood and throughout their adult lives”. Training is essentially delivered only from specialist surgical centres and lasts an indicative 5 years with each trainee satisfying an “Annual Review of Competence Progression”. The first 3 years are designed to provide broad general experiential and didactic learning of the speciality with exposure to all sub-speciality elements. Core syllabus is delivered through a rolling 3-year programme of National Training Days delivered across all United Kingdom centres (currently remotely) at which trainee attendance is compulsory. The last 2 years are composed of ongoing general paediatric cardiology and training in chosen “special interest, themed for service” areas. The trainee must demonstrate progression each year, which is assessed on the basis of an electronic portfolio of documented workplace-based assessments, logbook, certificates of attendance, audits, reflective practice, and anonymised and collated patient and colleague multisource feedback. There are also “multiple consultant reports” to be discussed with the trainee prior to each “annual review of competence progression”. Trainers or educational supervisors must meet with trainees multiple times each year. Their annual report is critical in determining whether the level of entrustment of the trainee is satisfactory for progression to the next year of training.

In other European countries, education in paediatric cardiology varies with specific countries’ regulations. Most countries have their national curriculum with a recommended structured training and subsequent evaluation and exam in paediatric cardiology, e.g., in the Netherlands and Germany. Consequently, European paediatric cardiology training is not uniform and even suffers from a lack of recognition as a sub-specialty in several countries. In Belgium, education in paediatric cardiology follows a period of 5 years of training in paediatrics. The sub-specialty cardiology training consists in general of a 3-year fellowship in a training centre during which the trainee receives exposure to out-patient clinics, pre- and post-operative paediatric cardiology care, echocardiography, cardiac catheterisation, and fetal cardiology. As there is no official training curriculum, the depth and breadth of clinical exposure vary, and there is no official evaluation of the quality and level of training in the specialty. Paediatric cardiologists remain registered as paediatricians. Given this example, which is similar in several other European countries, the Association for European Paediatric and Congenital Cardiology took the initiative to develop official recommendations on basic training in paediatric and congenital cardiology to ensure a high standard of education in the field with a view towards certification and eventual unification of European training.^17^ The recommendations cover a detailed list of knowledge, skills, and attitudes in the respective fields of the Association for European Paediatric and Congenital Cardiology working groups. Paediatric cardiology training builds upon a minimum of 3 years of paediatrics adding up to a total of 6 years of training.

“Exit” qualifications also vary significantly between different countries. In Ireland, trainees are evaluated annually during their training period by their trainers and senior academic supervisors and signed off by a committee, usually under the governance of the Royal College of Physicians, as having reached the sufficient standard of training. Log books or electronic portfolios typically record the required number of supervised echocardiograms, cardiac catheterisation procedures, electrophysiology procedures, and clinic attendances in addition to attendance at general paediatric cardiology conferences (e.g., British Congenital Cardiac Association or European conferences). A “Certificate of Satisfactory Completion of Specialist Training” is awarded. Many trainees will also choose to pursue a dedicated area of research, which usually results in the award of a Doctor of Medicine or Philosophy degree before, during (out of programme), or after their Certificate of Completion of Training. Certification of completion of specialist training is entered onto the Medical Register and is a requirement for substantive consultant appointment.

In the United Kingdom, there is no “exit” qualification as such. Trainees are assessed annually on their level of competence (soon to be “capability”) (Table 2). After the first 3 years of “core” training, it is expected that trainees will achieve Level 3 of all aspects of the curriculum. It is also expected that they will have scored >50% in the formative knowledge-based assessment, and failure to achieve this is an indicator of concern and the trainee may be offered additional help and or training time to achieve this before progression to their special interest programme. After the final fifth year, the trainees will be expected to achieve Level 4 in all generic capabilities and all sub-speciality areas except the most specialised technical procedures such as complex interventional or electrophysiological interventions for those training in such areas. “Sign off” for “Certificate of Completion of Training” requires a statement of trust from their educational supervisors, detailed and verified documentation such as log books and certificates of attendance/training, work-based assessments, satisfactory multisource feedback, and multiple consultant reviews (including from non-cardiologists) at the final “annual review of competence progression”. Annual review of competence progression recommendation is then passed to the General Medical Council for final approval and registration of Certificate of Completion of Training. Final certification also includes verification that the trainee has attended some management training and demonstrated evidence of reflective practice.

**Table 2.** Level of competence/capability.

Level	Descriptor
Level 1	**Entrusted to observe only** – no provision of clinical care
Level 2	**Entrusted to act with direct supervision**: The trainee may provide clinical care, but the supervising physician is physically within the hospital or other site of patient care and is immediately available if required to provide direct bedside supervision
Level 3	**Entrusted to act with indirect supervision**: The trainee may provide clinical care when the supervising physician is not physically present within the hospital or other site of patient care, but is available by means of telephone and/or electronic media to provide advice and can attend at the bedside if required to provide direct supervision
Level 4	**Entrusted to act unsupervised**

In Europe, a logbook to ensure the quality of the training is under development and intends to be compatible with the current national curriculum guidelines. The trainee will be evaluated by the local head of the training department. In future, it is proposed that a final written exam will have to be passed to become an Association for European Paediatric and Congenital Cardiology certified paediatric and congenital cardiologist. Many European trainees, especially those with an interest in non-invasive imaging, are taking the European Association of Cardiovascular Imaging echocardiography examination during their training period.

In the United States of America, paediatric cardiology trainees, having completed a paediatric residency, are able to sit for the Board Examination in paediatric cardiology under the American Board of Pediatrics at the end of their 3-year core cardiology training. Trainees meet regularly with fellowship directors during their training to review monthly rotation evaluations, 6-monthly or annual reviews (sometimes with a committee) to evaluate whether they are making progress in the six Accreditation Council for Graduate Medical Education core competencies (Table 3). A logbook is maintained outlining numbers of echocardiograms, cardiac catheterisations, and electrophysiology studies required to be performed or attended. The Board Examination in paediatric cardiology is a multiple-choice question format examination.

**Table 3.** Comparison of competency frameworks.

*General Medical Council (GMC)*
Professionalism
Good clinical care
Relationships with families and patients
Working with colleagues
Managing workplace
Social responsibility/accountability
*Accreditation Council for Graduate Medical Education in the USA (ACGME)*
Professionalism
Medical knowledge
Patient care
Practice-based learning and improvement
Interpersonal and communication skills
Systems based practice
*Canadian Medical Education Directives for Specialists (CanMEDS)*
Professional
Medical expert
Communicator
Collaborator
Manager
Health Advocate
Scholar

### Adult congenital heart disease

The number of adults with CHD has increased dramatically over the last two decades.^18^ Despite the presence of this growing population, knowledge and understanding of adult CHD remain poor amongst medical physicians and general cardiologists.^19,20^ This lack of knowledge has also been shown to negatively impact patient care.^20^ With only a small number of adult congenital heart disease trained specialists, educational opportunities are limited as not all cardiology residency or training programmes have access to a local expert.^21^ Some groups in the United Kingdom have created one or two-day courses in adult congenital heart disease in an attempt to fill such gaps in training. For United Kingdom trainees, more in-depth adult congenital heart disease training can be accessed as 1 or 2-year “special interest for service” modules from adult or paediatric cardiology training programmes. The e-learning website of the Adult Congenital Heart Disease Learning Centre (which has since evolved into a key component of the Heart University) was originally created to overcome these geographical and temporal constraints. The aim of the curriculum is to provide the minimum knowledge required by a physician to treat adults with congenital heart disease safely and appropriately. The syllabus of the material was designed by a working group of experts and consists of approximately 50 distinct modules, which cover all of the lesions seen in adults with congenital heart disease and the common medical topics encountered.

Accredited expertise in adult congenital heart disease requires specific formal training in a centre of excellence. In the United Kingdom, the remainder of Europe and North America, there is a general consensus that a training period of 24 months is recommended to complete full adult congenital heart disease sub-specialty training/fellowship.^22^ Trainees can enter from either an adult or paediatric cardiology background with regional and national variations existing in the proportion of candidates that enter from these two streams. Consequently, the two groups have obvious differences in knowledge and experience, and their differing training needs should be addressed by the local programme. In the United States of America, adult congenital heart disease training became standardised in 2015 under a dedicated 2-year curriculum. Once trainees have completed their adult congenital heart disease fellowship, they are eligible to take the adult congenital heart disease certification exam offered by the American Board of Internal Medicine. No formal examination in adult congenital heart disease exists in Ireland and the United Kingdom, but trainees, most commonly from an adult cardiology background, are typically evaluated throughout their training by their trainers and signed off in the same way as for paediatric cardiology.

While some uniformity is developing in regard to the duration and structure of training programmes, the nature of the training provided, the methods of continual assessment of the trainees, and the assurance of competency of the trainers remain highly variable. Some important concepts are evolving in each of these domains.

## Competency-based medical education

Traditionally, medical education was divided into didactic formal learning and an “expert-observed” apprenticeship for learning and acquiring skills. Given several weaknesses in this approach, McGaghie and colleagues provided an early description of competency-based medical education in 1978, in which they differentiated competency-based medical education from subject-oriented and integrated curricula through specific characteristics.^23^ Its organisation matched those functions required for medical practice, and medical trainees were required to master specific performance objectives, the acquisition of which could then be empirically tested.

“The intended outcome of competency-based medical education is a health-professional who can practice medicine at a defined level of proficiency, in accord with local conditions, to meet local needs.”^24^

Steps in development of competency-based education curriculum were quite specific^24,25^:

1. The identification of the core abilities required of trainees.
2. Explicit definition of the required competencies and their different components.
3. Definition of milestones along a developmental pathway for those same competencies.
4. The identification of educational activities and instructional methods.
5. The identification of different tools of assessment to gauge progress along those milestones.
6. The design of desired programmatic outcomes.

Competency-based medical education is now a widely recognised terminology in education, especially after the introduction of the Canadian Medical Education Directives for Specialists framework (CanMEDs) and the Outcome Project of the Accreditation Council for Graduate Medical Education (ACGME) in the United States.^26,27^

The CanMEDS physician competency framework is one of the clearest in describing seven roles for physicians, which are critical to the education of paediatric and congenital cardiologists: *medical expert, collaborator, communicator, leader, health advocate, scholar, and professional*.^28^ The overriding goal of competency-based medical education is maximising the care of patients through the development of a competent cardiologist vis-à-vis integration of all seven of these competencies.^24^ The implementation of competency-based medical education requires an organised and structured set of interrelated competencies termed a “competency framework”. The integration of competencies across congenital cardiology fellowship educational programmes and meaningful competency-based clinical supervision may be lacking in some jurisdictions. Many components of the desired competencies are common to the competency frameworks of Canadian Medical Education Directives for Specialists, Accreditation Council for Graduate Medical Education and General Medical Council (Table 3), but what do we make of the differences? Are these differences an important reflection of the training needs of differing systems, or are they unnecessary differences that undermine the uniformity of training and international collaboration? Indeed, a commonly cited frustration with competency-based medical education is lack of clarity in what it actually represents referring to it as “fuzzy thinking”. The “blurred language” around competencies, milestones, and activities can be confusing, and we need clear understanding and agreement among all stakeholders on what these terms represent.^29^ In the United Kingdom, this “phraseology” has evolved further due to “shape of training” and trainees are assessed as “capable” rather than “competent” which is a subtly different semantic, largely stemming from the perceived need to be “capable” of independent practice.

Nonetheless, the question must be asked as to whether trainers fully understand the competencies their trainees need to reach or whether there is a lack of understanding, primarily from a lack of faculty development in relation to the respective frameworks.^30^ How can we expect trainees to understand what is expected of them, if faculty lacks clarity in comprehending the expected competencies themselves? Only through such faculty development can trainers be equipped to provide adequate competency-based clinical supervision, thereby training fellows to provide safe patient care and competency-based performance. A further frustration to the complex language used around these concepts is the failure to translate what some deem relatively esoteric competencies into concrete clinical practice.

## Entrustable professional activities

Entrustable professional activities were developed to attempt to bridge such a gap between competency frameworks and clinical practice.^31^ These are the units of professional practice cardiologists use in their daily work.^32^ They tend to concentrate on clinical skills, but they also include the relevant knowledge, skills, attitudes, and professional roles. They may range in complexity from taking a cardiac history to performance of an echocardiogram, pericardiocentesis, or right heart catheterisation. Usually, entrustable professional activities are well-defined activities which are entrusted to appropriately trained personnel. The fundamental role for these entrustable professional activities is that they can be safely completed at the completion of training to ensure a safe competent practitioner. Assessment of entrustable professional activities therefore focuses on the ability to safely and competently complete such tasks. Other competencies, such as effective communication skills, professionalism, and skills associated with collaboration, are equally important requiring regular assessment. Each entrustable professional activity should be assessed several times, composed of different components and increase in complexity as trainee progresses through fellowship. Trainees progress on an entrustment scale from observer only to performance without supervision, level 1–4 (Table 2).

We need to have a frank conversation about which activities are critical in congenital cardiology. Do we assess every single professional activity encountered in clinical practice, each of which should be assessed several times, or do we adopt a more pragmatic approach and limit activities to more critical comprehensive tasks which translate across multiple different patients and different skillsets? How do we get the sweet spot between these approaches to serve our trainees best in reaching entrustment?

Another critical question is how do we, as trainers, reach decisions on entrustment? Is it always black or white that trainees reach level 4 entrustment in these activities or is this disingenuous given the potential for errors, uncertainty, or ambiguity in several clinical decision-making scenarios^33,34^? Is the need for crystallisation of competencies into practice sacrificing the nuances in real-world learning?

How we monitor entrustable professional activities and competencies is relevant and a matrix format that maps the important domains of the competence may prove helpful in visualising trainee progress (e.g., CanMEDS framework).^35^ Among cardiology trainees, entrustable professional activities may require several competencies, often requiring integration within the one task (e.g., taking a paediatric cardiology history may combine several domains of competence/capability).

Entrustable professional activities are now becoming a central component in training with the European Society of Cardiology incorporating them into their recent core curriculum document.^36^ Since all professional activities occur within a social setting, the entrustable professional activities defined within the document are aligned with the CanMEDS physician competency framework. Though the inclusion of entrustable professional activities is to be commended, they undoubtedly represent a significant logistical challenge in busy clinical cardiology departments with limited faculty.

## Educationalist training

Historically, many educationalists have promoted the use of problem-based learning in training. Problem-based learning originated from McMaster University in Canada and was then taken up by other educationalist centres in the Netherlands, before spreading further afield.^37,38^ Interestingly, there was a divergence in problem-based learning, with the McMaster group advocating a hypothetico-deductive approach compared to a constructivism approach adopted by the Maastricht group.^39^ In 1996, only seven recognised Master’s in Health Professions Education or Master’s in Medical Education programmes existed worldwide, which grew to over 76 programmes by 2012.^40^ Typically, the Master’s in Medical Education is a 2-year programme, with units including:

- curriculum development
- instructional design
- assessment
- leadership and management
- statistics
- research methodologies

These units are typically followed by a thesis on one region of interest. Some programmes are entirely online distance learning, while other programmes have a blended learning approach with online combined with summer residencies (3 weeks each year) in the university. This latter approach may facilitate greater interaction with international colleagues from different educational disciplines and specialities. Interestingly, the curriculum within these programmes evolve over time, with some (School of Health Professions Education, Maastricht University, Maastricht, Netherlands) adopting a more bespoke curriculum focusing on:

- coach-led educational design
- leadership
- research through self-regulated learning
- development of competencies using rich formative feedback

Coaching, both in the moment and overtime, overseen by a competence committee is fast becoming a model adopted by some educational groups, including the Royal College of Physicians and Surgeons of Canada (www.royalcollege.ca). Coaching should aim to foster a longitudinal trusting relationship between trainer and trainee, set out specific learning and performance goals, employ rich formative feedback, and ultimately promote self-directed learning.

## Curriculum development

The curriculum (as distinct from the syllabus, which is a document detailing the specifics of the course) is at the core of training of congenital cardiologists, and although often quite variable in structure between different countries and continents, it usually covers all the fundamental components of ambulatory cardiology (outpatient cardiology), preventive cardiology, non-invasive imaging, cardiac catheterisation, electrophysiology, heart failure, and transplantation. Development of a curriculum is very time-consuming and requires input from multiple stakeholders including faculty but, most importantly, the trainees themselves. A six-step approach has been advocated^41^:

- problem identification
- general and targeted needs assessment
- setting of goals and objectives
- formulating educational strategies
- implementation evaluation
- feedback

Each of these issues can be seen on a continuum known as the SPICES model^42^:

- student/teacher-centred
- problem-based/information-gathering
- integrated/discipline-systematic
- community-based/hospital-based
- elective/uniform
- systematic/apprenticeship-based

Newer programmes trend towards the left side of the spectrum while more established programmes trend towards the right of the spectrum. This model could prove helpful in developing but also revising curricula for different cardiology programmes.

The curriculum must be a living document and evolve or adapt to changes within the practice of congenital cardiology. The developments within adult congenital heart disease, fetal cardiology, cardiovascular genetics, and artificial intelligence point to the need for adaptability within the structure of congenital cardiac curricula. Increasingly, many departments are cognisant of this importance of a spiral curriculum^43^ or “spiral of learning”, where trainees iteratively revisit topics of learning, deepening their understanding of the area of interest with each revisit.^44^ The “hidden curriculum” often encompasses what is implicitly learned, professionalism, and empathy for patients, rather than simple transmission of knowledge and skills.^45^

## The concept of the master adaptive learner

The ultimate aim of the curriculum is to provide our trainees with the knowledge, skills, and attitudes to become an expert in congenital cardiology. To achieve this goal, educators aim to promote a novel concept of the master adaptive learner, “who utilizes the metacognitive approach to self-regulated learning that leads to adaptive expertise development”.^46^ Or in plain English, the master adaptive model of learning provides a framework for self-regulated learning that integrates all domains of learning, evolves with time and expertise, and persists throughout a lifetime of work.^47^ It seeks to lead trainees away from superficial learning *towards deep human learning*, which is defined as higher-order cognitive understanding of complex concepts of a specific condition, including aspects of ambiguity and uncertainty related to the condition, as opposed to superficial learning, which is learning facts or rote-learning.^48^ Deep human learning also involves critical analysis of new ideas, linking them to previously known concepts and meta-cognitive thinking (reflection) to promote long-term understanding.

## Simulation

**Figure 1.**
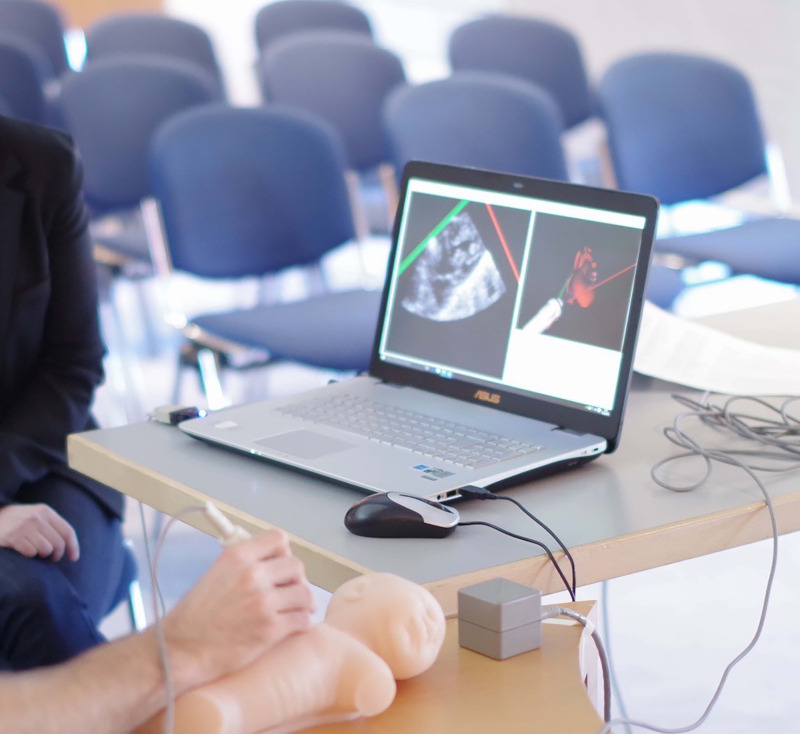
Simulation in transthoracic echocardiography. Reproduced with permission from EchoCom GmbH. Nieheim, Germany.

**Figure 2.**
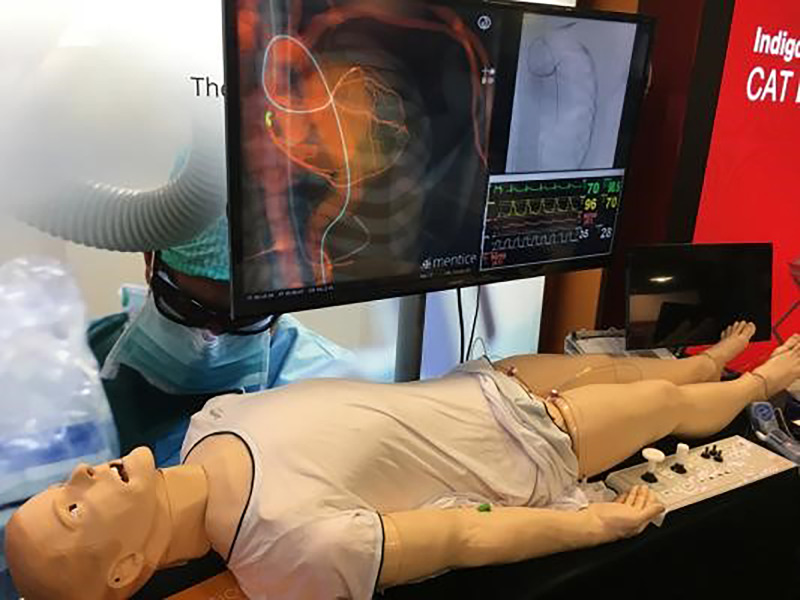
Simulation in cardiac catheterisation by Mentice. Reproduced with permission from DAIC, photograph by Dave Fornell.

A powerful tool to promote deep human learning is simulation, which has seen increased adoption over the last decade.^49^ Increased demands on training hours for trainees and greater focus on patient safety have prompted greater incorporation of simulation within the standardised curriculum. The development of simulation and its incorporation into the curriculum require significant time and resources. There is a significant workload setting up the simulator, maintaining high-fidelity simulation, and training of faculty in the management of simulation.^50^ Feedback and debriefing, deliberate practice, and curriculum integration are all critical to reaping the benefits from simulation. Each cardiology department will decide based upon their curriculum the degree of mastery learning, the different ranges of difficulty, integrating clinical variation, and individualised learning. Simulation-based healthcare education has great potential from undergraduate education, training cardiology fellows or indeed in continuing medical education. Greater application of simulation will inevitably become integrated into curricula in non-invasive imaging (Fig 1), cardiac catheterisation (Fig 2), and electrophysiology training^51,52^; and, while speculative, we envisage that physical simulators may be replaced by virtual-reality-based simulation, as has developed in other industries.

## Education in low resource settings

While many developments in education and training may be rapidly adopted in well-resourced settings, special consideration should be made for those training to practise in low resource settings.^53^ Even with the increasing number of formal training programmes in various low- and middle-income countries globally, a critical shortage remains of specialists trained to care for patients with congenital heart disease.^54^ Furthermore, those training in high-income countries and returning to practice in a low resource setting are not uncommonly confronted with clinical scenarios they may not have anticipated, nor were trained to manage.^55^ The significance of this shortage of congenital heart disease specialists in low resource settings is critical, because over 90% of the children in the world with congenital heart disease are born in low- and middle-income countries.^56^ While additional barriers contribute to the persistent inequities in utilisation of congenital heart disease resources and interventions in low resource settings, the establishment and maintenance of formal training programmes in congenital heart disease remain a significant barrier.^57^ In addition, some high-income countries may also have low resource settings where there are limited cardiology services, so-called underserved areas.

Notwithstanding such challenges that exist in low resource environments, it is important to recognise that a number of paediatric cardiologists are being trained entirely within these countries and are now looking to serve their respective populations. With widespread availability of online material, and easy access to this material, few barriers remain to acquiring theoretical foundations. However, it remains a fact that structured fellowship training in an academic institution that systematically instils strong foundational understanding and clinical skills, sharpens critical thinking, and fosters contextually relevant research is perhaps the best route to all around development.^56^ A severe shortage of such academic institutions exists within low resource environments, which remains one of the main reasons why trainees continue to apply for fellowships in academic institutions in higher income countries.

The challenge now lies in working towards creating a framework for training in paediatric cardiology and congenital cardiac care that can leverage global expertise and make it available for the programmes in low- and middle-income countries. In addition to the unique disease profile in low resource regions, often resulting from late presentation, and co-morbidities that include infections and under-nutrition, this training must look to equip the trainees with the diverse knowledge and skill sets that are necessary to deliver care specific to the needs of their home countries and build comprehensive capacity for the future (Table 4). It is very enlightening to compare and contrast training in different low resource settings across the globe.

**Table 4.** A framework for acquisition of specific skills and attributes for a paediatric and congenital cardiologist in low-resource environments.^78^

Skills and attributes needed	Reasons	Training elements
Empathy and insight	Enables thoughtful and contextually appropriate management strategies recognising the challenges that individual families face	Experience in outreach clinics or primary care facilities remote from tertiary centres; case studies that explore socio-economic challenges
In-depth understanding of regional disease profile	Available knowledge sources from high-income regions do not cover specific challenges encountered in low-income regions	Structured course content that specifically includes clinical challenges from low-resource environments
Multi-tasking	Sub-specialised paediatric cardiac care is expensive; acquiring multiple skills is feasible and enables cost-effective care	In-depth exposure to all dimensions of paediatric cardiac care; clinical, imaging, intensive care, catheter interventions, and electrophysiology; focus on acquisition of strong theoretical foundations
Commitment to quality improvement (QI)	Low-cost QI measures such as infection control have to be embraced and implemented to ensure patient safety and good outcomes	Formal training in foundations of quality improvement science; participation in multi-institutional QI databases (such as IQIC); participation in regular audits
Teaching and training skills	Education and training other healthcare professionals enables capacity building and essential for sustainability	Frequent structured presentations during the training programme to hone teaching and communication skills
Research	Research is integral to generating contextually relevant information that can guide local practices	Training in research methodology, mandated postgraduate research thesis

IQIC = International Quality Improvement Collaborative for Congenital Heart Disease (https://iqic.chboston.org/).

### Training in India

Paediatric cardiology was only identified as a distinct specialty in India in 2000. Up until then, there were no structured programmes in existence, and all paediatric cardiac care was delivered by a handful of adult cardiologists with a strong interest in paediatric cardiology. At that time, there were very few institutions with focused expertise in the care of children with heart disease, with infant and newborn heart surgery undertaken in only five institutions. However, the declining infant mortality from readily preventable causes unmasked a massive burden of congenital heart disease and underscored the need to build capacity in paediatric cardiac specialties.^58^

With the advent of select institutions established over the last 20 years, an increasing number of programmes are now delivering consistently good outcomes in specialised paediatric cardiac care, including infant and newborn heart operations. The establishment of the Pediatric Cardiac Society of India in 1997 also provided paediatric cardiac specialties with a distinct identity.

Two parallel training streams exist now for paediatricians to train in paediatric cardiology. The majority of the country’s paediatric cardiologists have been trained in a fellowship programme in one of the 11 hospitals that are accredited by the National Board of Examinations. This was initially started as a 2-year fellowship (Fellowship of the National Board) and then evolved into a 3-year postgraduate degree (Diplomate of the National Board). Over 100 paediatric cardiologists have been trained through this path and they constitute the majority of the country’s pediatric cardiology workforce. More recently, another training stream, a 3-year Doctorate of Medicine degree has been introduced by the Medical Council of India and is available in three academic university hospitals with an annual intake of six candidates. Both these streams require the candidates to pass a structured exit examination for the degrees to be awarded. Despite these developments, a number of challenges remain including the shortfall of training programmes and other care providers (paediatric cardiac surgeons and intensive care experts).

### Training in South Africa

In South Africa, paediatric cardiology training and indeed, medical education are largely based on a historically United Kingdom–like system, with the MBChB degree, six initial years of training, and a series of junior training posts prior to sub-specialisation. Over the last decade, the imperative of decolonisation of training curricula has resulted in a context-specific training schedule, with community and rural service built into training. Paediatric cardiologists are fully trained paediatricians (usually 3–4 years), who then undergo a 3-year training period at a public service academic institution with a logbook and curriculum-based training. This is followed by a formal exit examination comprising a two-part written and rigorous oral examination. The outcome qualification is a “Certificate in Paediatric Cardiology”, awarded by the College of Medicines of South Africa. Fully funded training places are competitive; however, training posts are also provided for international colleagues with external funding, and thus the first paediatric cardiologists in Namibia, Malawi, and many other parts of Africa have received their training in South Africa. What is currently lacking in the country are formal training mechanisms for those wishing to gain further qualifications. Most elect to pursue these internationally. Finally, it is also strongly encouraged that fellows undertake research projects and obtain either a Master’s in Medicine (newly compulsory for paediatricians) or a Master’s of Philosophy (recommended for paediatric cardiologists) during this time.^59-61^

### Training in Brazil

In Brazil, formal training in paediatric cardiology comprises a 2-year medical residency programme, funded by the Ministry of Education, with ambulatory cardiology (in and out-patients), emergency and critical care, non-invasive imaging, and cardiac catheterisation as minimal requirements. Certified paediatricians or cardiologists may apply, and selection is through a written test, dispensed by the residency boards of each state. A national board also assesses and certifies the institutions that meet the requirements to provide such training. Currently, there are 27 certified institutions in the country. After finishing the initial training, most residents will embark on one to two optional years to improve or develop skills in specific areas, such as echocardiography or adult congenital heart disease. Some will pursue further training in partner institutions within and outside Brazil. As there are few available positions for the nationally funded programme, some institutions offer non-residency training positions. However, trainees must undergo similar selection criteria, working hours, academic and clinical activities as residents. Finally, to become certified as a paediatric cardiologist, all residents or trainees must pass a national theoretical and practical test, developed jointly by the Brazilian paediatric and cardiology societies.

## Interdisciplinary education

In addition to consideration of global congenital cardiology training paradigms, modern healthcare, irrespective of jurisdiction, necessitates a multi- and inter-disciplinary team approach, and this requirement is typically exemplified in the care of the patient with congenital heart disease.^62^ The care team often includes, but is not limited to:

- congenital cardiologists and its various subspecialties (i.e., non-invasive cardiac imager, interventionalists etc.)
- cardiothoracic surgeons
- cardiac anaesthesiologists
- intensivists
- perfusionists
- cardiovascular geneticists

Interdisciplinary education calls for a larger worldview and includes the broad gamut of allied healthcare professionals^63^:

- nursing
- dieticians
- speech and language therapists
- physical and occupational therapists
- respiratory therapists
- ultrasonographers
- pharmacists

Students in each healthcare area should be exposed to all other disciplines during their training. If students work together at all levels of training and are taught by faculty members from multiple disciplines, the integration of interdisciplinary care will be enhanced. In addition to learning to work together as team members, students in different programmes can learn from one another as well.

While true interdisciplinary education remains somewhat elusive, it places the well-being of the patient at the centre of its paradigm and surely is the way of the future. Such interdisciplinary education will be facilitated by technology enhanced learning and the development of multidisciplinary educational forums that are open-access to all components of the healthcare team, such as webinars and other digital learning platforms.

## Technology enhanced-learning

With increasing interest in technology enhanced learning, trainees are sourcing information directly from the internet. Although some outstanding material is available, it is difficult to navigate the multiple portals to access this information. Furthermore, despite the presence of multiple outstanding educational opportunities, several are outdated, poorly produced, or simply misleading. Consequently, while much has occurred in other domains of internet access (e.g., shopping portals, music portals, etc.), properly curated and regularly updated speciality-specific educational portals are needed that guide the learner to the “best-in-class” educational materials. For example, Heart University, a new e-learning website about paediatric and congenital cardiac care, has recently harnessed this interest by developing a carefully curated open-access library of educational material for all providers of paediatric and congenital cardiac care, not only for trainees but also for practicing providers.^57^ The site is managed and curated by Editorial Boards for the component sites (*Pediatric Cardiac Learning Centre, Adult Congenital Heart Disease Learning Centre*) that are comprised of an international group of experts covering a wide range of subspecialties, with endorsement from major international organisations in the fields of paediatric and congenital cardiac care. The Heart University platform was used to promote this new form of congenital cardiac education, with additional marketing via e-mail and social media. While this open-access online platform can never replace the hands-on clinical learning necessary to develop comprehensive competence in paediatric and congenital cardiac care, it can play an important role in supplementing such learning. Even more, it has the potential to address some of the educational barriers present in many low resource settings.^55,56^

## Assessment

**Figure 3.**
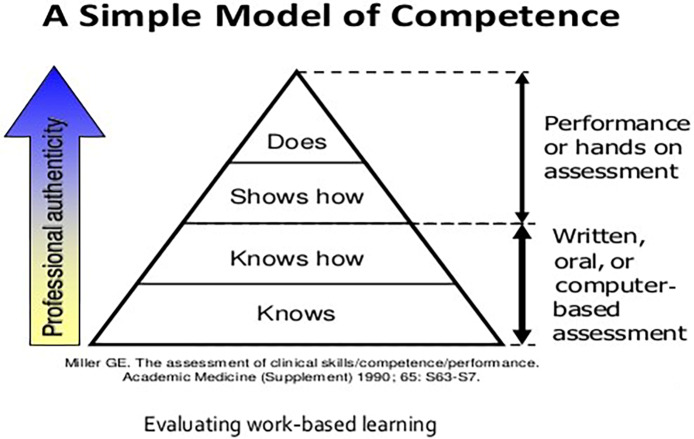
Miller’s pyramid of competence.

No dialogue on education and training would be complete without discussion of the critical nature of assessment in driving learning. The degree and extent of assessment vary markedly between different programmes and systems and over different time frames. In past decades, formalised feedback between trainer and trainee was a rarity. With the development of formalised training programmes, feedback was required on at least an annual basis from the fellowship director. Additionally, individual faculty may provide feedback on how well a task has been performed such as clinical evaluation in the outpatient department, an echocardiogram, or performance of a right heart catheterisation. Miller’s pyramid of competence highlights the importance of the trainee “doing” the task (Fig 3). Even now, such assessments are highly variable in their structure and frequency, and only recently has feedback from trainee to trainer been facilitated and encouraged. Although continual assessment should clearly play a major role in establishing competency to practice, the final assessment in some countries is the summative assessment of Board Certification Examination, a high-stakes assessment, based upon multiple-choice questions. It is important to ask whether this form of assessment is optimal for trainees or whether better alternatives exist or could be developed?

An alternative new approach to assessment has emerged which has been adopted by some countries (e.g., United Kingdom) similar to programmatic assessment.^64^ Programmatic assessment is a unique strategy whereby data about the competence and progress of the fellow are collected throughout their training fellowship, analysed, and where needed, complemented with purposively collected additional assessment information. These data can then be used to inform the cardiology fellow and their trainer of their progress and facilitate high-stakes decisions at the end of their fellowship training.^65^ Typically, a variety of assessment instruments are used, with a greater variety of tools (e.g., including annual reviews of competence performance, work based assessments, multiple consultant reports, capabilities and statement of levels of attainment) providing richer insights into the progress of the trainee (Table 5).^66,67^ Programmatic assessment is being used in various medical school settings around the world and some cardiology programmes, and it is also becoming more popular in graduate medical education and continuing professional development.^68,69^

**Table 5.** Assessment tools of trainees.

Multiple-choice questions^79^
Oral examination^80^
Objective structured clinical examination (OSCE)^81^
Case-based short essay^82^
Workplace-based assessments (WBA)^83^
Procedure-based assessment (PBA)
Case-based discussion (CBD)
Clinical evaluation exercises (CEX)^84^
Direct observation procedural skills (DOPS)^85^

In the programmatic assessment “approach”, each assessment should provide meaningful rich feedback to the trainee.^67^ Formative feedback is most effective when the trainee has a growth mindset, receives the feedback in an appropriate setting and time, and there is a trusting relationship established between the trainer and trainee.^70,71^ These individual assessments should not be used for high-stakes decision-making, but rather their function is to facilitate the fellow to visualise their performance, develop specific learning goals, and then successfully reach them.^66^ Individual assessments are accumulated into a portfolio, which can be subsequently reviewed by a faculty member or committee to create an overall picture, facilitating high-stakes decisions.^67^ By using this programmatic assessment approach, all information about a trainee can be reviewed by an assessment committee and aid in reaching summative decisions about the progress of the trainee through fellowship training.^69^ Combining multiple different assessment instruments may provide an optimal evaluation of training fellows.^70^ It is worth noting that assessments can be routinely integrated into the everyday care of the patient in the outpatient department, reviewing an electrocardiogram or echocardiogram. and they do not always mandate a formalised setting.^72^ If the trainee is failing to meet required standards, supportive solutions can be offered. Ongoing conversations between the trainee and trainer will help ensure the overall personal development of the trainee. Rather than the traditional pass–fail decision, programmatic assessment focuses not just on the attained competence levels, but equally important, on the developmental process of the trainee in reaching that level of competence.^64^ One of the benefits of this approach is that it reflects how we learn as we progress throughout our careers in postgraduate practice after graduation from fellowship.

## Medical education in the post COVID-19 world

The COVID-19 pandemic has resulted in a major re-organisation of healthcare which has translated to a profound impact on how training can be delivered.^73^ This transformation is witnessed through the rise of the webinar, with greater interest and indeed appreciation of the power of online e-learning formats. Indeed, the first webinar organised by Heart University for its “Contemporary Questions in Congenital Heart Disease” webinar series, which began in May 2020, was attended by 1374 live audience attendees from 100 countries spanning six continents. This webinar was the largest gathering of providers of paediatric and congenital cardiac care outside of the Quadrennial World Congress of Pediatric Cardiology and Cardiac Surgery, with similar attendance recorded for their subsequent monthly webinars. Even more impressive have been the number of registered website users who have subsequently viewed the recordings made freely available on the website following the live event, demonstrating the thirst for enduring educational material. Other webinar series will mean that the worldwide access to high-quality online education will be unprecedented in the years to come. Such webinar series include:

- those organised by Heart University
- those organised by “Congenital Heart Academy”
- those organised by “World University for Paediatric and Congenital Heart Surgery”
- a fellow training series organised by the Association for European Paediatric and Congenital Cardiology (via the Heart University portal)
- the conversion of several sub-specialty meetings to “virtual” from in-person

This “enforced evolution” is timely, as increasingly prior to the pandemic, physicians, allied healthcare providers, and trainees globally have been unable to attend high-quality educational conferences or other educational opportunities in person. The aetiology of this challenge is multi-factorial, with reasons including

- geopolitical changes (limited or delayed visa availability, entry exclusion)
- financial constraints
- time constraints related to commitments to patient care and reduced institutional study leave

This newly popular medium for online educational events helps to address many of these barriers and makes this educational material accessible to a global population despite these constraints.^74^ While the webinar will never fully replace the in-person conference, the level of participation observed surely raises questions as to whether it should play a more significant role in “sustainable academia” once the pandemic abates, especially when one considers its additional environmental benefits.^75^ Webinar fatigue due to potential over-exposure or cognitive overload also needs to be guarded against.^76^ Of note, the last Association for Medical Education in Europe conference was held through video-conferencing, where attendees attend as avatars in a virtual conference setting.

## Future directions for development of medical education within paediatric cardiology and adult h congenital heart disease

A number of steps may facilitate further development of medical education within our field:

- Although competency-based education has been widely adopted over the last two decades, we must reflect on how well it delivers for trainees. The language around competency-based medical education is challenging, and consistent clarity in what defines competencies, milestones, and entrustable professional activities is crucial. Given the lack of uniformity across different competency frameworks and clear gaps between competencies and clinical practice, we are at a critical inflection point where entrustable professional activities are being adopted into congenital cardiology training. Some countries are even moving beyond competencies to “capabilities” (“Shape of Training” in the United Kingdom), a subtle distinction of the critical importance of level 4 entrustment and the capacity for independent practice.
- There is wide variation in training standards and qualifications between different countries. Further efforts to standardise training standards, curricula, assessments, and certification would prove helpful although the specific competencies required for individual countries and their respective local populations need to be respected also.
- An honest appraisal of the design of the current curriculum and instructional techniques in training fellows may result in a shift from didactic-type teaching to a problem-based learning format or at least consideration of a hybrid of both systems. Variation theory strategies in training design can facilitate transfer of training in specific skills.^77^
- Implementation of simulation, where feasible financially and logistically, within the fellowship training programmes may see benefits in terms of echocardiography, cardiac catheterisation, and electrophysiology training. Although simulators pose a significant financial outlay and time investment, an agile solution for trainees in low and middle-income countries could be greater use of virtual reality headsets (e.g., Oculus rift) which are becoming cheaper and more widely available.
- Assessment *for* learning should focus on formative assessment with multiple assessment points utilising different tools of assessment.^65^ Programmatic assessment provides a high-quality method of holistic assessment of trainees, despite the effort involved in providing this level of assessment. What is clear is that timely and honest assessment drives learning, and insufficient or low-quality assessment fails to facilitate learning.
- Greater provision of repeated rich formative feedback to trainees within a coaching framework which promotes self-regulated learning should be encouraged.
- Greater work is needed to develop and support fellowship training programmes in low- and middle-income countries including realistic deliverables given limited resources. Technology enhanced learning (e-learning, webinars, and online academies) can be leveraged as a low-cost and easily accessed pedagogical tool to assist such low- and middle-income countries. Twinning fellowship programmes between low and middle-income countries with high-income countries would facilitate greater learning from both sides including rich insights into maximising outcomes in resource-limited environments.
- Increased resources need to be allocated to train adult congenital cardiologists given the number of paediatric cardiology patients transitioning to adulthood. Innovative solutions may need to be found to meet this impending manpower shortage.
- We would also advocate for a medical educational stream and sessions within major medical conferences, including those devoted to paediatric and congenital cardiac care.
- Finally, educational articles in congenital cardiology will provide an outlet for peer-reviewed high-quality research on medical education. *Cardiology in the Young* has fostered a progressive approach, agreeing to publish one or two articles in each journal issue dedicated to research and innovation related to medical education in the domain of paediatric and adult congenital cardiac care.

## Limitations

It is not possible for us to comment on training and education in every jurisdiction in the world, and we are well aware of the absence of representation from several countries. Furthermore, even within those jurisdictions discussed, there may be local imperatives that require customisation of training to meet the needs of the system and patient need. Nonetheless, this paper is one of the first to start a conversation on several important issues in medical education that we believe are relevant to all medical systems.

## Conclusion

In conclusion, this paper reviews the current status of training within paediatric and adult congenital cardiology. While we have seen some laudable developments, multiple opportunities exist for improvement and optimisation. Indeed, this article highlights the potential for further advances in education related to paediatric and congenital cardiac care, including:

- further developments in curriculum
- adoption of entrustable professional activities to bridge the gap between competencies and clinical practice
- enhancements in instructional design (technology enhanced learning)
- improvement in the strategies for assessment for learning
- greater use of repeated rich formative feedback and coaching
- harmonisation of training standards across different jurisdictions while fulfilling the competencies required to meet the needs of the local population

The speed of evolution within medical education and training must be matched by our ability to adapt to those changes, most recently exemplified by the rise of e-learning precipitated by the COVID-19 pandemic. Most critically, our role as educators remains to ensure that trainees are well trained with the capacity for self-regulated learning and critical reasoning, which will serve both themselves and their patients well throughout their careers. Hopefully this paper will help foster a dialogue between all the stakeholders involved in congenital cardiology training and education to further develop strategies and models in a concerted effort to realise that vision.
